# Osteogenic Potential of Multipotent Adult Progenitor Cells for Calvaria Bone Regeneration

**DOI:** 10.1155/2016/2803081

**Published:** 2016-04-28

**Authors:** Dong Joon Lee, Yonsil Park, Wei-Shou Hu, Ching-Chang Ko

**Affiliations:** ^1^Oral and Craniofacial Health Sciences Research, School of Dentistry, University of North Carolina, Chapel Hill, NC 27599-7455, USA; ^2^Department of Chemical Engineering and Materials Science, University of Minnesota, Minneapolis, MN 55455-0132, USA; ^3^Department of Orthodontics, School of Dentistry, University of North Carolina, Chapel Hill, NC 27599-7454, USA

## Abstract

Osteogenic cells derived from rat multipotent adult progenitor cells (rMAPCs) were investigated for their potential use in bone regeneration. rMAPCs are adult stem cells derived from bone marrow that have a high proliferation capacity and the differentiation potential to multiple lineages. They may also offer immunomodulatory properties favorable for applications for regenerative medicine. rMAPCs were cultivated as single cells or as 3D aggregates in osteogenic media for up to 38 days, and their differentiation to bone lineage was then assessed by immunostaining of osteocalcin and collagen type I and by mineralization assays. The capability of rMAPCs in facilitating bone regeneration was evaluated* in vivo* by the direct implantation of multipotent adult progenitor cell (MAPC) aggregates in rat calvarial defects. Bone regeneration was examined radiographically, histologically, and histomorphometrically. Results showed that rMAPCs successfully differentiated into osteogenic lineage by demonstrating mineralized extracellular matrix formation* in vitro* and induced new bone formation by the effect of rMAPC aggregates* in vivo*. These outcomes confirm that rMAPCs have a good osteogenic potential and provide insights into rMAPCs as a novel adult stem cell source for bone regeneration.

## 1. Introduction

For the repair of large bone defects, a bone tissue engineering strategy has to employ osteogenic cells, osteoinductive growth factors, and osteoconductive scaffolds. The key to the success of this strategy is the effectiveness of the cell source. An ideal cell source for bone tissue engineering should have readily available cells, high proliferative potential* in vitro*, and a high osteogenic differentiation potential under* in vitro* culture condition. Various stem cells have been explored as the cell source for tissue engineering applications as they can be expanded and differentiated in culture to meet the demand [1–3]. In particular, adult stem cells are attractive because they can be isolated from patients, and autologous applications are possible. Their differentiation potential in bone lineage makes them promising cell sources in repairing skeletal defects caused by trauma, tumor removal, and congenital malformations [4, 5]. Currently, bone marrow derived mesenchymal stem cells (BMSCs), adipose tissue stem cells (ASCs), amniotic stem cells (ASCs), and multipotent adult stem cells (MAPCs) have been shown to possess osteogenic potential, and their potential use in bone regeneration has been explored [6–9].

Among those adult stem cells, BMSCs have been the most commonly used in bone tissue engineering. In the studies of other types of adult stem cells for bone regeneration, BMSCs have often been used as the control for comparison [10]. However, MSCs usually have less proliferative potential compared to pluripotent stem cells such as embryonic stem cells and induced pluripotent stem cells. Their expansion and differentiation potential may vary depending on the age of donor or patient. Many have reported the aging effects of donor source for MSC, and this effect may have implications in the potential use of autologous MSCs for bone regeneration [11, 12].

MAPC was first isolated from adult bone marrow during the subculturing of mesenchymal cells by Jiang et al. [13, 14]. Within the BMSC population, there was a group of cells that were identified to have an extensive expansion capability with high expression of Oct4, a pluripotent marker. MAPCs do not form teratoma when transplanted into mice and require no feeder layers. They can differentiate into various specialized cell types including mesodermal cells, muscle cells, endothelial cells, liver cells, and neuroectodermal cells under defined experimental conditions. Their wide range of differentiation potentials makes them attractive as a possible cell source for the regeneration of bone tissue. Recently, it was discovered that rMAPCs are similar to rat blastocyst-derived extraembryonic endoderm precursor (rXENP) cells, which showed extensive extraembryonic endodermal differentiation [15]. Under rMAPC culture conditions, rat hypoblast stem cells (rHypoSCs) are similar to rMAPCs and MAPC medium-shifted rXENP cells in their gene expression profiles and developmental potentials. rHypoSCs were derived from rat blastocysts in a more direct and rapid way assigning the lineage identity to rMAPCs, indicating that rMAPCs are originated by environmental reprogramming [16, 17].

Only a few studies have explored the osteogenic differentiation potential of rMAPCs; Ferreira et al. reported that titanium enriched hydroxyapatite scaffold could enhance bone regeneration in calvarial defect sites, and three-dimensional cell seeding method could also help rMAPCs to regenerate bone defects* in vivo* [18]. Inspired by the study of Ferreira et al. on three-dimensional (3D) culture method of rMAPCs, rMAPCs were also examined for their osteogenic differentiation potential as 3D aggregates [19]. In a recent publication, higher bone regeneration was induced by using a novel biomaterial, hydroxyapatite-gelatin calcium silicate (HGCS), with rMAPCs in a rat model. In the study, HGCS had an osteogenic effect on rMAPCs and stimulated calvarial bone regeneration [20].

Recent studies on immunological characteristics of human multipotent adult stem cells (hMAPCs) revealed that they have comparable immunomodulatory effects to human mesenchymal stem cells (hMSCs) [21]. MHC class I was expressed highly in MSCs but only at a low level in MAPCs. Both hMSCs and hMAPCs do not express MHC class II on the surface. hMAPCs showed strong immunosuppressive effects on T-cell proliferation and were not influenced by MHC compatibility [22]. These findings have clinical relevance of MAPCs for the potential use in bone regeneration. In humans, while hMAPCs can expand over 70 population doublings, hMSCs can only have 20 to 25 population doublings [23]. This extensive growth capability of hMAPCs allows cell banking for better cell supply and quality control. The immunomodulatory property of hMAPCs may allow them to be a universal donor.

In this study, we hypothesized that both rMAPCs in the form of 3D aggregates and single rMAPCs can have osteogenic differentiation potential after osteogenic differentiation, and the rMAPCs can stimulate* in vivo* bone formation. To test the hypothesis, we examined the osteogenic potential of rMAPCs in monolayer and 3D aggregate culture to determine whether the rMAPCs have* in vitro* osteogenic differentiation potential or not. The 3D aggregates were further assessed for their* in vivo* osteogenic potential in bone regeneration.

## 2. Materials and Methods

### 2.1. Preparation of HG Scaffolds

The preparation of hydroxyapatite and gelatin (HG) scaffolds and HG coated dishes was described in a previous study [20]. Briefly, HAP-Gel slurry was biomimetically synthesized by the coprecipitation method using* in situ* hybridization of calcium silicates or titanium oxide with HG powders. The powders of calcium hydroxide and HG were mixed and cross-linked with enTMOS (bis[3-(trimethoxysilyl)-propyl]ethylenediamine) for 30 seconds before adding a calcium chloride solution to the mixture. As the mixture thickened, the material was quickly transferred into 1 cc syringes with 1 mm inner diameter needles, and the material was extruded to make intertwined structures with macropores. The structures were then dried for 7 days and sterilized with cold ethylene oxide (EO) gas before use.

### 2.2. MAPCs and Differentiation in 2D and 3D Aggregates

The isolation and culture of rMAPCs from rat have been described in previous studies [13, 14, 18]. For the 2D monolayer cultures, rMAPCs (8 × 10^4^ cells) were seeded with growth media (GM) in 24-well plates (Costar, Corning Inc. Life Sciences, Lowell, MA, USA). Cells were allowed to grow for 5 days until they reach 80 to 90% of confluency. Subsequently, growth media were replaced by osteogenic media (OM, Dulbecco's Modified Eagle's Media, 10% Fetal Bovine serum, 0.2 mM *β*-glycerophosphate, 0.2 *μ*M ascorbic acid, and 10 nM Dexamethasone). The media were changed every 3 days for up to 42 days.

To prepare the 3D aggregates, rMAPCs (2 × 10^3^ cells) were seeded in suspension with rMAPC growth media in 96-well rounded bottom ultralow attachment plates (Costar, Corning Inc. Life Sciences, Lowell, MA, USA), using a modification of a forced aggregation method reported previously [24–26]. Briefly, rMAPC suspension was centrifuged at 1400 rpm for 4 min to allow cells to form aggregates over time. Aggregates were grown for 5 days. Then, ten aggregates were distributed to each well of 24-well ultralow attachment plates (Costar, Corning Inc. Life Sciences, Lowell, MA, USA) and switched to osteogenic media. Fresh osteogenic media were supplied every 3 days up to 38 days to stop the differentiation.

### 2.3. Characterization for rMAPCs

A characterization study of rMAPCs was described in previous studies [13, 14, 18]. rMAPCs were provided by Dr. Hu from the University of Minnesota. Briefly, a monolayer culture of rMAPCs was harvested by trypsinization and suspended in phosphate-buffered saline (PBS) with 3% serum at 100,000 cells per Eppendorf tube (1.5 mL). All immunostaining (both osteocalcin and collagen type I) was performed for the rMAPCs on the HG coated dish or the frozen section of rMAPC aggregates except for Oct4 and CD31. Both rMAPCs and 3D aggregates were fixed with 4% paraformaldehyde (PFA), rinsed, treated with 0.3% H_2_O_2_ for 30 minutes, dehydrated in 100% methanol, rehydrated with water, and transferred to Triton X-100 in PBS solution. Then, avidin/biotin activity was blocked with Avidin-Biotin kit (Dako, CA, USA) and the sample was rinsed three times with PBST (Triton X-100 in PBS) and specific antibody binding sites were blocked for 30 min with 0.4% fish skin gelatin in PBS. Cell/matrix layers were incubated overnight at 4°C with rabbit primary antibody against rat collagen type I (NB600-408, Novus Biologicals, CO), rinsed three times, and incubated with secondary biotinylated goat anti-rabbit IgG antibody (NB730-B, Novus Biologicals, CO) for 30 min at room temperature (RT). The cell/matrix layer was incubated in ABC complex (Vector Laboratories, CA) according to the manufacturer's protocol and rinsed three times, and DAB Chromogen solution (Liquid DAB+Substrate Chromogen System, Dako, CA) was added to the matrix layer for 5 to 20 minutes until a brown color developed. MC3T3-E1 cells were used as positive control and negative controls were without primary antibody.

rMAPCs morphology on the HG coated dish was examined using a scanning electron microscope (SEM) (Hitachi TM3000). rMAPC cultures were fixed in 4% PFA at room temperature (RT) and then analyzed at 15 kV in low vacuum state for nonconductive materials. The HG coated dishes with rMAPCs were cut, embedded in resin blocks, sliced into ultrathin sections with a diamond knife, and stained with a saturated solution of uranyl acetate in methanol, followed by Reynold's lead citrate, and the image was acquired with a Hitachi H-7000 TEM at 120 kV.

### 2.4. Proliferation of rMAPCs

rMAPCs (P18) were plated in 96-well plates at a density of 2 × 10^3^ cells per each well using basal osteogenic media. The proliferation of the rMAPCs in growth and osteogenic media was conducted by MTS assay following the company's instruction. Composition and method to prepare growth and osteogenic media for rMAPCs were outlined in the previous publications [18]. The MTS (3-(4,5-dimethylthiazol-2-yl)-5-(3-carboxymethoxyphenyl)-2-(4-sulfophenyl)-2H-tetrazolium (Promega Co., Madison, WI, USA)) reacted with cells at 37°C for 1 hour. After transferring the solution into a 96-well plate, absorbance of growth and osteogenic media group was measured on days 1 and 7, at 490 nm using a plate reader (Bio-Rad, Hercules, CA, USA). The proliferation of 3D aggregates was described in a previous study [19].

### 2.5. Osteogenic Differentiation of Single rMAPCs and 3D Aggregates

For the 2D monolayer cultures, rMAPCs (8 × 10^4^ cells) were seeded with growth media in 24-well plates (Costar, Corning Inc. Life Sciences, Lowell, MA, USA). Cells were allowed to grow for 5 days until they reached 80 to 90% of confluency for mesodermal differentiation. Then, growth media were replaced by osteogenic media. Media were changed every 3 days. rMAPCs were differentiated in osteogenic media for 3, 7, 14, 21, and 42 days.

To prepare the 3D aggregates, rMAPCs (2 × 10^3^ cells) were seeded in suspension with rMAPC growth media in 96-well rounded bottom ultralow attachment plates (Costar, Corning Inc. Life Sciences, Lowell, MA, USA), using a modification of the forced aggregation method reported previously [24–26]. Briefly, MAPC suspension was centrifuged at 1400 rpm for 4 min to allow cells to form aggregates over time. Aggregates were grown for 5 days. Then, ten aggregates were distributed to each well of 24-well ultralow attachment plates (Costar, Corning Inc. Life Sciences, Lowell, MA, USA) and switched to osteogenic media. Fresh osteogenic media were supplied every 3 days up to 38 days for osteogenic differentiation.

### 2.6. Analysis of Single rMAPCs and 3D Aggregates for OCN and Col-1 and Mineralization

For immunostaining of Col-I and OCN, single rMAPCs and cryosectioned 3D aggregates were fixed with 4% PFA, rinsed, incubated for 30 min with 0.3% H_2_O_2_ in 100% methanol, rehydrated, and transferred to PBS-Triton solution. After blocking for 30 min with 0.4% fish skin gelatin in PBS, both cells and aggregates were incubated overnight at 4°C with rabbit primary antibody against rat collagen type I (NB600-408, Novus Biologicals, CO), rinsed thrice, and incubated with secondary biotinylated goat anti-rabbit IgG antibody (NB730-B, Novus Biologicals) for 30 min at RT. After incubation in ABC complex (Vector Laboratories, CA) following the manufacturer's instruction, DAB Chromogen solution (Dako, CA) was added for 5–20 minutes until a brown color developed. For the OCN, primary OCN antibody (Santa Cruz) and FITC conjugated secondary antibody (Abcam) were used instead. Images were acquired with a Nikon fluorescence imaging system and a DP70 color digital camera equipped with color image software (DP11, Olympus USA, Center Valley, PA, USA).

Both single rMAPCs on HG coated dish and 3D aggregates were fixed in neutral buffered 10% formalin, and then the aggregates were embedded in optimal cutting temperature (OCT) solution. Five-micrometer sections were cut and stained with Alizarin Red S (ARS) solution (pH 4.2) to observe mineralized extracellular matrices. Mineralization on HG coated dishes by rMAPCs was also stained with ARS on days 3, 7, 14, and 21. After drying, stained dishes were scanned for the images acquisition.

### 2.7. *In Vivo* Implantation of MAPC Aggregate and HG Scaffolds

Differentiated rMAPC aggregates in osteogenic media were seeded onto the HGCS scaffolds (*n* = 3). Two test groups (HG only and HG+MAPCs) were used with three rats in each group for a total of six male Sprague-Dawley rats (Charles River, Wilmington, MA; about 250 to 300 g, 7 weeks). An 8 mm, critical-sized defect (CSD) was created after anesthetization by Ketamine-HCl injection (10 mg/kg: Putney Inc., Portland, ME, USA). Three rats were implanted with HG scaffold only and the other three rats were implanted with HG scaffold and MAPC aggregates. For the mineral apposition rate (MAR) measurement, fluorochrome labels, Alizarin Red S (30 mg/kg, Sigma-Aldrich, St. Louis, MO, USA), and Calcein (20 mg/kg, Sigma-Aldrich, St. Louis, MO, USA) were injected perivascularly into each animal twice during the study. Alizarin Red S was administered 10 days after the surgery and Calcein was given 15 days before sacrifice. The interlabeling periods were 10 and 70 days.

### 2.8. Micro-CT Analysis

After 12 weeks, the calvariae were removed and trimmed by preserving the implanted sites before fixing in 10% formalin for 7 days at 4°C. Then, the specimens were preserved in 70% isopropyl alcohol at 4°C. The calvaria explants were scanned by using a micro-CT system (mCT 40; Scanco Medical, Brüttisellen, Switzerland) at 70 kV and 114 mA with a 200 ms integration time. Detailed setting parameters for acquisition and analysis of the acquired images were described in a previous study [20].

### 2.9. Fluorescence Microscopy for Mineral Deposition

The detailed method for the slide preparation was described previously [22]. The fluorescent images of completed sections were acquired by using a fluorescence microscope (Eclipse Ti-U, Nikon Instruments Inc., Melville, NY, USA) with bright field, TRITC and FITC filters, and a digital camera. After fluorescent image acquisition, calvaria specimen slides were further stained with Steven Blue by counterstaining with Van Gieson to visualize the formation of newly formed bone (NFB) tissue for the quantitation as previously described [19]. Briefly, entire images of the medial (central) sagittal histologic section were acquired with a DP70 color digital camera equipped with color image software (DP11, Olympus USA, Center Valley, PA, USA) under 20x magnification and then merged using Adobe Photoshop CS6 (Adobe Systems Inc., San Jose, CA, USA) to recreate as one figure. The new bone surface area (B.Ar.) and the total area of each defect (T.Ar.) were measured in pixels by using an automated image analysis system (ImageJ software version 1.46R, NIH, Bethesda, MD, USA) to calculate the NFB (in %: B.Ar./T.Ar/0.01) based on the standardized protocols of the American Society for Bone and Mineral Research [27]. A one-tailed Student *t*-test was used to compare the means between the groups.

### 2.10. Statistical Analysis

All results were quantified as mean ± standard deviation. ANOVA was used to define whether differences between each group were significant or not. When the *p* value was less than 0.05, the differences were considered significant.

## 3. Results and Discussion

For effective bone regeneration using a tissue engineering and regenerative medicine strategy, the stem cells and the scaffold material are the two major components. Because the osteogenic inducing capability of the scaffold materials was already examined in previous studies [18–20], our study was mainly focused on the rMAPCs for their* in vitro* osteogenic differentiation and* in vivo* bone forming potential. The detailed mechanisms by which a component (either stem cell or material) contributes to bone regeneration are not known. Besides, the fate of implanted stem cells and the extent of their direct contribution to bone regeneration remain controversial [28].

**Figure 1:**
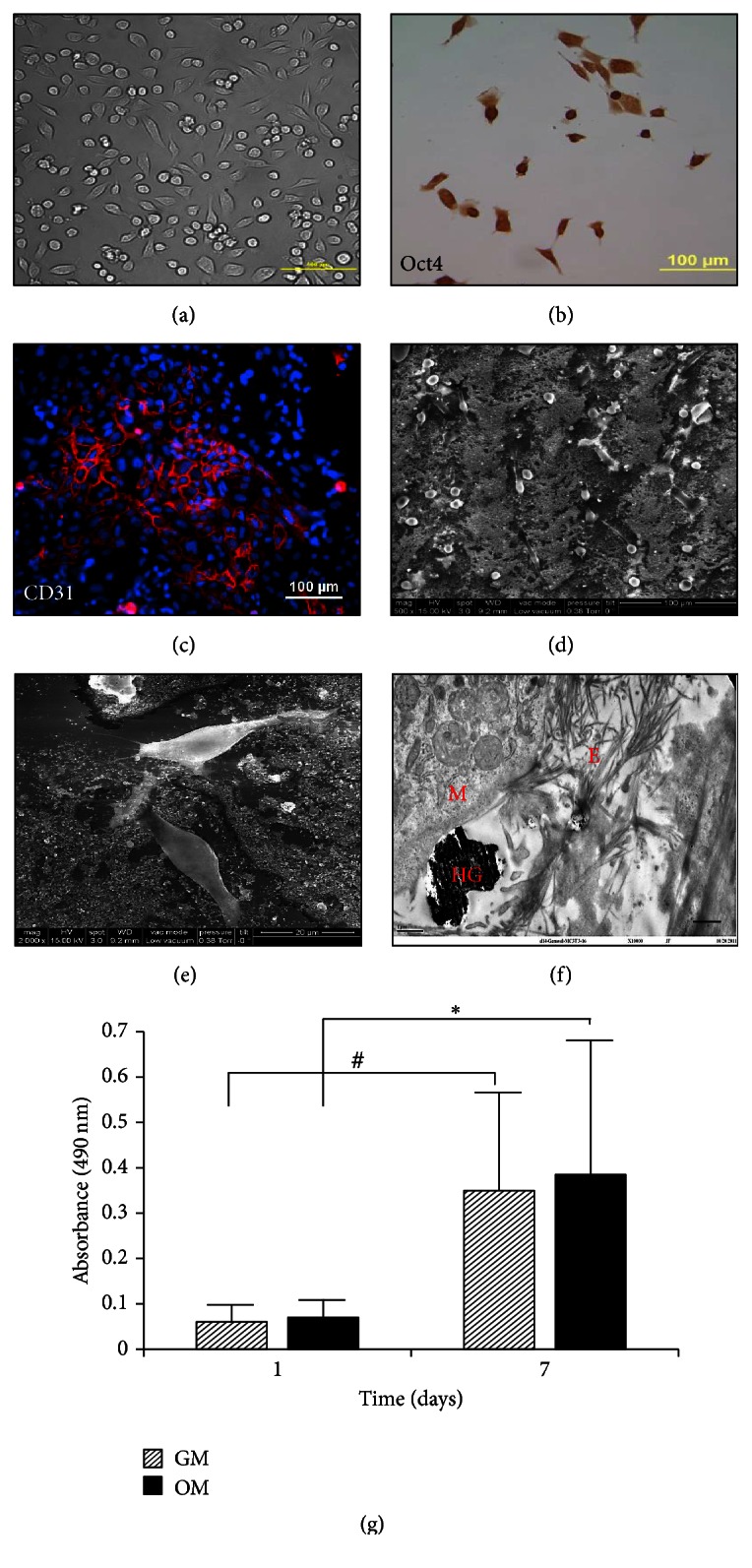
Characterization of rat MAPCs. DIC microscopic picture of MAPCs culture on the fibronectin coated dish (a). MAPCs were stained with Oct4 antibody conjugated with chemiluminescent dye (b) and CD31 antibody conjugated with TRITC (c). SEM analysis visualized MAPCs on the HG materials in low power (d) and high power (e). TEM image of MAPCs that interacted with ECM and HG materials (f): hydroxyapatite (HG), MAPCs (M), and extracellular matrix (E). Scale: 5 *μ*m. MAPCs proliferation was assessed at baseline (within 24 h after seeding) and after 7 days in osteogenic media; ^*∗*, #^ *p* < 0.05.

rMAPCs are small, elongated, or oval shape cells when grown on the fibronectin coated culture dishes (Figure 1(a)). rMAPCs express a high level of the transcription factors Rex1 [29] and Oct4 [30], which are pluripotency markers for stem cells. In this study, undifferentiated rMAPCs were confirmed by showing the expression of Oct4 and CD31 by immunostaining (Figures 1(b) and 1(c)).

rMAPCs can be differentiated into endothelial, adipogenic, chondrogenic, and osteogenic linage cells, cardiomyocytes, and hepatocytes [13, 31]. In 2D tissue culture plates, rMAPCs do not attach strongly like other mesenchyme origin cells (Figures 1(a) and 1(b)). The morphology of undifferentiated rMAPCs in HG was examined by SEM (Figures 1(d) and 1(e)) and TEM (Figure 1(f)). After 2 days, rMAPCs were weakly attached to the surface of HG compared to the cells on fibronectin coated plates. Most attached cells exhibited round or oval shapes rather than flat ones.

To observe* in vitro* proliferative potential, rMAPCs were tested in either growth or osteogenic medium for MTS assay. In general, when higher numbers of rMAPCs react with MTS, higher formazan activity will be yielded. Using the biochemical reaction, we measured MTS activity of the rMAPCs on days 1 and 7. At day 1, rMAPCs cultured in growth medium had an OD value of 0.06 ± 0.07 and 0.07 ± 0.08 in osteogenic media. The low value of OD is due to the nature of the rMAPC culture, maintaining low cell density to keep the cells undifferentiated. On day 7, rMAPCs in growth and osteogenic media had ODs of 0.35 ± 0.44 and 0.39 ± 0.6, respectively (Figure 1(g)). Both growth and osteogenic media stimulated rMAPC proliferation. In many previous studies, MSCs were used as control to compare the growth potential with new adult stem cell source. However, the differentiation potential of MSCs is largely dependent on the age of the donor so it is recommended to maintain MSCs at low passage number (passage 6 or less for human cells) for their stemness and high proliferative potential [21]. While rMAPCs share characteristics of pluripotent stem cells with high expression of Oct4, rMAPCs can be expanded to more than 70 doublings.

**Figure 2:**
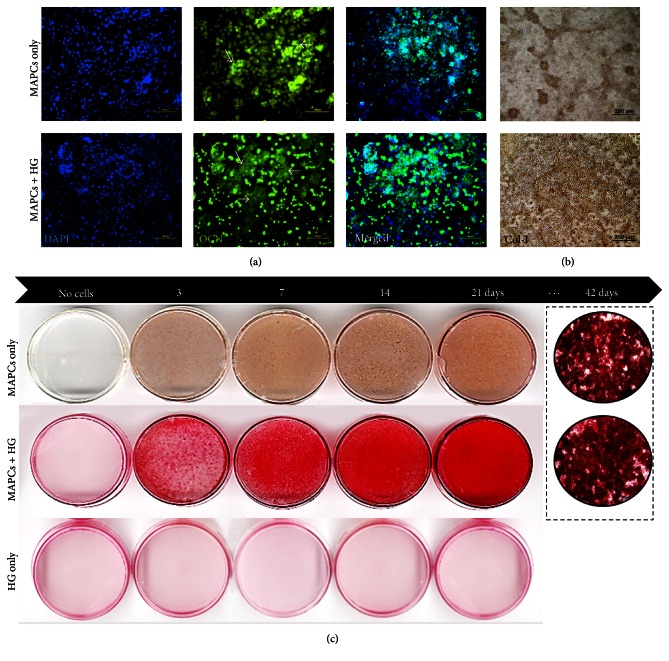
*In vitro* assessment of osteogenic differentiation of MAPCs on the HG material-coated dishes. Immunohistochemical staining with osteocalcin (a) and collagen type I (b) antibodies against MAPCs cultured on the HG coated culture plate (bottom) and without a coating as control (top). Mineral formation was detected by Alizarin Red S staining (c) after culturing MAPCs with osteogenic media on the coated dish (middle), no coating as control (top), and coated dish without MAPCs (bottom). Microscopic images further confirmed the ability of mineralization by MAPCs after 42 days of osteogenic differentiation.

Differentiated cells were immunostained for osteocalcin (OCN) and collagen type I (Col-I) after 21 and 38 days of osteogenic differentiation in 2D and 3D culture, respectively. Osteocalcin, generated by mature osteoblasts, is known to deposit onto the extracellular matrix as an indicator of bone repair [32]. Osteocalcin was detected around and between each differentiated cell (Figure 2(a), white arrows). It appears to show expression of OCN when rMAPCs were differentiated alone (Figure 2(a), top) and also when rMAPCs were differentiated on the HG coated dish (Figure 2(a), bottom). Although OCN expression was not quantified, rMAPCs in osteogenic media could express higher level than that in growth media. rMAPCs under growth media did not express OCN in both control and coated dish (data not shown). The expression of Col-I on differentiated rMAPCs showed a similar trend to OCN expression on the differentiated rMAPCs. HG coating increased the expression level of Col-I in osteogenic differentiation of rMAPCs (Figure 2(b)).

**Figure 3:**
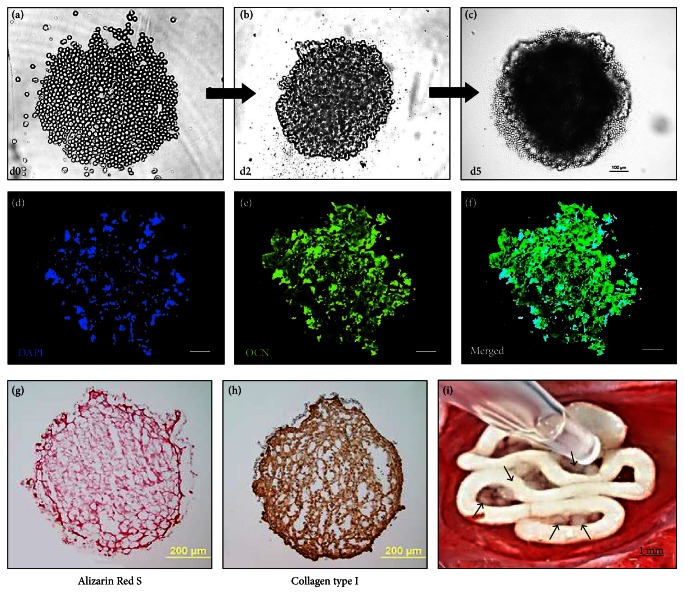
Characterization of osteogenic differentiation of MAPCs in 3D aggregate culture. The generation of MAPC aggregates was observed on 0, 2, and 5 days after suspension culture ((a) to (c)). Cell aggregation formed a compact spheroid at day 5 (c). After 28 days, osteogenic potential of 3D aggregates was analyzed by immunostaining with osteocalcin ((d), (e), and (f)) and collagen type I (h) antibodies. Mineralization was also observed by Alizarin Red S staining (g). (i) showed direct aggregates seeding on the scaffold during surgery on rat calvarial defect (arrows indicate 3D aggregates). Scale bars: 100 *μ*m ((a) to (f)), 200 *μ*m ((g) and (h)), and 1 mm (i).

Although we did not quantify the expression level of osteogenic protein and mineralization to compare between 2D and 3D differentiation, we did observe that both single rMAPC culture and 3D aggregate showed clear expression of OCN and Col-I protein as well as mineral formation (Figures 2 and 3). Another advantage for using rMAPC aggregate is to increase the cellular viability during the seeding process by sustaining the aggregates in the defect site without absorption (Figure 3(i)). While seeding single cells on the scaffold or defect site, cells can be easily lost by their absorption into the surrounding tissues. Generally, a scaffold plays a role as a carrier to prevent the loss of cells during the seeding process. Still, the poor attachment characteristic makes single rMAPCs even difficult to be successfully retained in the scaffold.

The mineralization of rMAPCs differentiated in a monolayer was tested by staining the calcium deposition after 7, 14, and 21 days of osteogenic differentiation. Mineral deposition in the control plate without HG coating increased over time up to 21 days (Figure 2(c), top) and was visible 7 days after differentiation. Differentiation in the control plate (rMAPC only) showed more intense mineralization by ARS staining than the HG coated dish without rMAPCs (Figure 2(c), bottom). Differentiated aggregates were also tested for the same markers and mineralization as the cells in 2D culture. The* in vitro* osteogenic differentiation study indicated that rMAPCs in both 2D and 3D culture system could differentiate into osteogenic cells. Although the osteogenic effect by HG materials on rMAPCs was not prominent in the 2D culture system, still they may have a critical influence on bone regeneration. Further studies will be needed to provide clarification.

**Figure 4:**
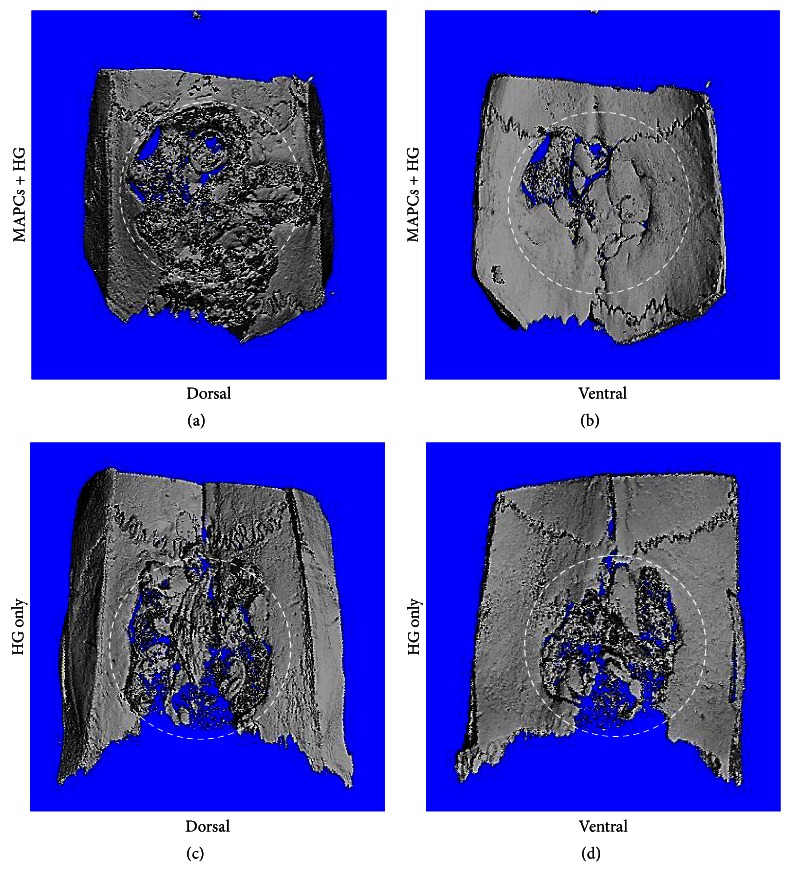
Micro-CT images of critical-sized calvarial defects after 12 weeks of implantation. White dotted circle represents defect site (8 mm in diameter). MAPCs with HG group ((a) and (b)) and HG-only group ((c) and (d)).

To evaluate whether the bone regeneration potential rMAPC aggregates can stimulate bone formation, the rMAPC aggregates were tested by depositing on HG scaffolds in calvarial critical-sized defects in a rat model. After 12 weeks of implantation, calvariae were resected and evaluated by *μ*CT (Figure 4). The resection procedure must be carried out with great caution because any small errors in the defect site can cause significant misinterpretation of the result. In Figure 4, the HG scaffold with MAPC aggregates regenerated bone in the defect site better than HG scaffold only. Many times, the micro-CT image from the dorsal and ventral sides showed different degrees of bone formation. Zooming on the ventral side of the calvaria clearly showed that higher bone regeneration was induced by rMAPC aggregates. One possible prediction is that many rMAPC aggregates seeded on the defect site flowed through the HG scaffold to the bottom of the defect site. Without absorption, the 3D aggregates could stimulate the bone regeneration mostly on the dorsal site. Most of all, the regenerated site with rMAPC aggregates with HG had a continuous bridge through the center of the defect. This demonstrated a high degree of bone regeneration in the calvarial defect site and is used as a scoring system by many researchers [33]. On the other hand, bone regeneration on the defect site with HG showed less bone formation and a more disintegrated appearance than HG with rMAPC aggregates.

**Figure 5:**
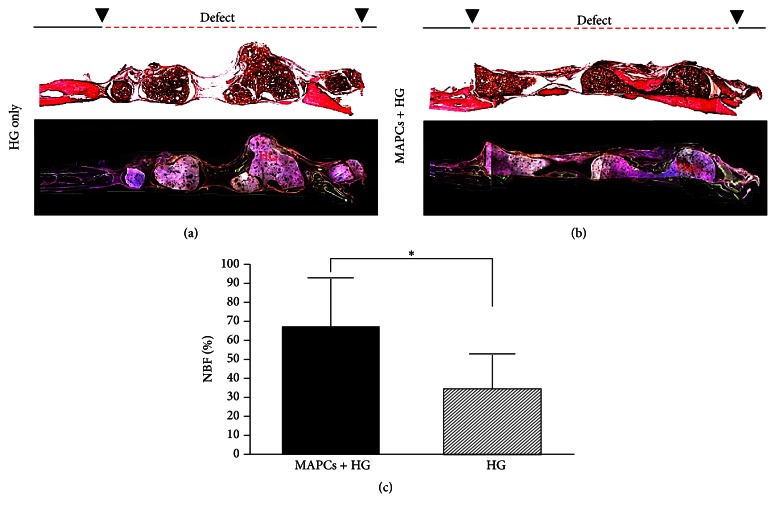
Sagittal section of the defect area after 12 weeks of implantation. Sections were stained with Steven Blue and Van Gieson ((a) and (b), top) and fluorescence image was labeled with Calcein and Alizarin Red S dye ((a) and (b), bottom). The area of new bone formation (NFB) was quantified in % using ImageJ software; ^*∗*^ *p* < 0.05 (c).

For the analysis of new bone formation, micro-CT image alone is inadequate to distinguish new bones from the surrounding host bone or the radiopaque HG materials. Histological and fluorescent image can provide further information on new bone formation. Undecalcified calvaria sections were stained with Steven Blue and Van Gieson to identify new bone formation (brighter red) in the defect site (Figures 5(a) and 5(b)). New bone formation was calculated to be 66.99 ± 26.44% for rMAPCs with HG group and 34.44 ± 19.62% for the HG-only group (Figure 5(c)). As the higher percentage indicated, more new bones were observed in the center of defect apart from host tissue in rMAPCs with HG group (Figure 5(b)). Unlike the rMAPCs with HG group, HG-only groups relied on the cell source from the host bone. Most of the new bone was observed near each end (near host bone) without much new bone formation in the center of the defect area (Figure 5(a)). However, whether implanted rMAPCs did differentiate into osteoblasts* in vivo* to create new bone is not clear. There is also a possibility that rMAPCs could induce more repairing capability of host cells by extending its coverage to the center of the defect although rMAPCs themselves did not generate bones. Further studies using a cell tracking method will be necessary to find whether rMAPCs can directly participate in bone regeneration.

Taken together, rMAPC aggregates can be differentiated into osteogenic linage* in vitro* both in 2D monolayer and in 3D aggregate culture systems.* In vivo* bone regeneration using rMAPC aggregates with HG scaffold resulted in higher bone regeneration than using HG scaffold only. This supports our hypothesis that MAPCs can differentiate into osteogenic cells and also promotes bone regeneration.

For possible improvement in bone regeneration using rMAPCs in the future, a study on the mechanism of rMAPC aggregated in terms of osteogenic signal pathways such as Wnt and TGF beta signal transduction may provide crucial factors to modulate the osteogenic differentiation of rMAPCs. Although rMAPCs in this study showed an osteogenic potential both* in vitro* and* in vivo*, whether their bone regenerative capability is superior to other types of adult stem cells is still uncertain. Therefore, a comparative study of rMAPCs with other types of stem cells will also give a better insight into choosing the right cell source for future bone tissue engineering applications.

## 4. Conclusion

The findings in this study support the osteogenic potential of rMAPCs and the direct effect on bone regeneration both* in vitro* and* in vivo*. rMAPCs showed a good cell proliferation ability in both growth and osteogenic media. Also,* in vitro* osteogenic differentiation was able to be induced in osteogenic media for 28 days and confirmed by expression of osteogenic markers, osteocalcin, and collage type I. rMAPCs formed the moderate mineralization up to 21 days and further differentiation up to 42 days clearly showed the deposition of highly mineralized extracellular matrix by differentiated cells. After examining rMAPCs in 3D aggregate culture in osteogenic media for 39 days, high level of osteocalcin, collagen type I, and mineralization was observed. The 3D aggregates of rMAPCs showed a significantly higher level of osteogenic differentiation outcome than rMAPCs in 2D monolayer culture* in vitro*. Thus, aggregates were carried by HG scaffold in a construct and implanted into the rat calvarial defect. The* in vivo* osteogenic effect of rMAPCs with HG scaffolds was revealed by the superior bone formation on the defect site. Micro-CT, histology, and histomorphometric analysis also showed much higher bone formation for the group implanted with rMAPC aggregates than HG scaffold only.

In summary, the outcomes confirmed that rMAPCs have superior osteogenic potential in the application of 3D aggregates for both* in vitro* mineralization and* in vivo* bone formation. These results may regard the rMAPCs as a novel adult stem cell source for the future clinical applications in bone regeneration.

## References

[1] Liao Y. H. T., Verchere C. B., Warnock G. L. (2007). Adult stem or progenitor cells in treatment for type 1 diabetes: current progress. *Canadian Journal of Surgery*.

[2] Mimeault M., Hauke R., Batra S. K. (2007). Stem cells: a revolution in therapeutics—recent advances in stem cell biology and their therapeutic applications in regenerative medicine and cancer therapies. *Clinical Pharmacology & Therapeutics*.

[3] Christoforou N., Gearhart J. D. (2007). Stem cells and their potential in cell-based cardiac therapies. *Progress in Cardiovascular Diseases*.

[4] Mauney J. R., Volloch V., Kaplan D. L. (2005). Role of adult mesenchymal stem cells in bone tissue-engineering applications: current status and future prospects. *Tissue Engineering*.

[5] Triffitt J. T. (2002). Stem cells and the philosopher's stone. *Journal of Cellular Biochemistry*.

[6] Wang X., Wang Y., Gou W., Lu Q., Peng J., Lu S. (2013). Role of mesenchymal stem cells in bone regeneration and fracture repair: a review. *International Orthopaedics*.

[7] Pipino C., Pandolfi A. (2015). Osteogenic differentiation of amniotic fluid mesenchymal stromal cells and their bone regeneration potential. *World Journal of Stem Cells*.

[8] Tsuji W., Rubin J. P., Marra K. G. (2014). Adipose-derived stem cells: implications in tissue regeneration. *World Journal of Stem Cells*.

[9] La Francesca S., Ting A. E., Sakamoto J. (2014). Multipotent adult progenitor cells decrease cold ischemic injury in *ex vivo* perfused human lungs: an initial pilot and feasibility study. *Transplantation Research*.

[10] Peister A., Woodruff M. A., Prince J. J., Gray D. P., Hutmacher D. W., Guldberg R. E. (2011). Cell sourcing for bone tissue engineering: amniotic fluid stem cells have a delayed, robust differentiation compared to mesenchymal stem cells. *Stem Cell Research*.

[11] Raggi C., Berardi A. C. (2012). Mesenchymal stem cells, aging and regenerative medicine. *Muscles, Ligaments and Tendons Journal*.

[12] Jones D. L., Rando T. A. (2011). Emerging models and paradigms for stem cell ageing. *Nature Cell Biology*.

[13] Jiang Y., Jahagirdar B. N., Reinhardt R. L. (2002). Pluripotency of mesenchymal stem cells derived from adult marrow. *Nature*.

[14] Jiang Y., Jahagirdar B. N., Reinhardt R. L. (2007). Pluripotency of mesenchymal stem cells derived from adult marrow. *Nature*.

[15] Debeb B. G., Galat V., Epple-Farmer J. (2009). Isolation of Oct4-expressing extraembryonic endoderm precursor cell lines. *PLoS ONE*.

[16] Lo Nigro A., Geraerts M., Notelaers T. (2012). MAPC culture conditions support the derivation of cells with nascent hypoblast features from bone marrow and blastocysts. *Journal of Molecular Cell Biology*.

[17] Binas B., Verfaillie C. M. (2013). Concise review: bone marrow meets blastocyst: Lessons from an unlikely encounter. *Stem Cells*.

[18] Ferreira J. R., Padilla R., Urkasemsin G. (2013). Titanium-enriched hydroxyapatite-gelatin scaffolds with osteogenically differentiated progenitor cell aggregates for calvaria bone regeneration. *Tissue Engineering Part A*.

[19] Ferreira J. R., Hirsch M. L., Zhang L. (2013). Three-dimensional multipotent progenitor cell aggregates for expansion, osteogenic differentiation and ‘*in vivo*’ tracing with AAV vector serotype 6. *Gene Therapy*.

[20] Lee D. J., Padilla R., Zhang H., Hu W.-S., Ko C.-C. (2014). Biological assessment of a calcium silicate incorporated hydroxyapatite-gelatin nanocomposite: a comparison to decellularized bone matrix. *BioMed Research International*.

[21] Jacobs S. A., Roobrouck V. D., Verfaillie C. M., Van Gool S. W. (2013). Immunological characteristics of human mesenchymal stem cells and multipotent adult progenitor cells. *Immunology and Cell Biology*.

[22] Jacobs S. A., Pinxteren J., Roobrouck V. D. (2013). Human multipotent adult progenitor cells are nonimmunogenic and exert potent immunomodulatory effects on alloreactive T-cell responses. *Cell Transplantation*.

[23] Roobrouck V., Clavel C., Jacobs S. A. (2011). Differentiation potential of human postnatal mesenchymal stem cells, mesoangioblasts, and multipotent adult progenitor cells reflected in their transcriptome and partially influenced by the culture conditions. *Stem Cells*.

[24] Subramanian K., Park Y., Verfaillie C. M., Hu W. S. (2011). Scalable expansion of multipotent adult progenitor cells as three-dimensional cell aggregates. *Biotechnology and Bioengineering*.

[25] Ng E. S., Davis R. P., Azzola L., Stanley E. G., Elefanty A. G. (2005). Forced aggregation of defined numbers of human embryonic stem cells into embryoid bodies fosters robust, reproducible hematopoietic differentiation. *Blood*.

[26] Subramanian K., Geraerts M., Pauwelyn K. A. (2010). Isolation procedure and characterization of multipotent adult progenitor cells from rat bone marrow. *Methods in Molecular Biology*.

[27] Parfitt A. M., Drezner M. K., Glorieux F. H. (1987). Bone histomorphometry: standardization of nomenclature, symbols, and units. Report of the ASBMR Histomorphometry Nomenclature Committee. *Journal of Bone and Mineral Research*.

[28] Salgado A. J., Coutinho O. P., Reis R. L. (2004). Bone tissue engineering: state of the art and future trends. *Macromolecular Bioscience*.

[29] Ben-Shushan E., Thompson J. R., Gudas L. J., Bergman Y. (1998). Rex-1, a gene encoding a transcription factor expressed in the early embryo, is regulated via Oct-3/4 and Oct-6 binding to an octamer site and a novel protein, Rox-1, binding to an adjacent site. *Molecular and Cellular Biology*.

[30] Scholer H. R., Hatzopoulos A. K., Balling R., Suzuki N., Gruss P. (1989). A family of octamer-specific proteins present during mouse embryogenesis: evidence for germline-specific expression of an Oct factor. *The EMBO Journal*.

[31] Park Y., Subramanian K., Verfaillie C. M., Hu W. S. (2010). Expansion and hepatic differentiation of rat multipotent adult progenitor cells in microcarrier suspension culture. *Journal of Biotechnology*.

[32] Chenu C., Colucci S., Grano M. (1994). Osteocalcin induces chemotaxis, secretion of matrix proteins, and calcium-mediated intracellular signaling in human osteoclast-like cells. *The Journal of Cell Biology*.

[33] Spicer P. P., Kretlow J. D., Young S., Jansen J. A., Kasper F. K., Mikos A. G. (2012). Evaluation of bone regeneration using the rat critical size calvarial defect. *Nature Protocols*.

